# Research on Face Image Digital Processing and Recognition Based on Data Dimensionality Reduction Algorithm

**DOI:** 10.1155/2021/3348225

**Published:** 2021-12-20

**Authors:** Ming He

**Affiliations:** Institute of Design, Chongqing Industry Polytechnic College, Chongqing 401120, China

## Abstract

Because face recognition is greatly affected by external environmental factors and the partial lack of face information challenges the robustness of face recognition algorithm, while the existing methods have poor robustness and low accuracy in face image recognition, this paper proposes a face image digital processing and recognition based on data dimensionality reduction algorithm. Based on the analysis of the existing data dimensionality reduction and face recognition methods, according to the face image input, feature composition, and external environmental factors, the face recognition and processing technology flow is given, and the face feature extraction method is proposed based on nonparametric subspace analysis (NSA). Finally, different methods are used to carry out comparative experiments in different face databases. The results show that the method proposed in this paper has a higher correct recognition rate than the existing methods and has an obvious effect on the XM2VTS face database. This method not only improves the shortcomings of existing methods in dealing with complex face images but also provides a certain reference for face image feature extraction and recognition in complex environment.

## 1. Introduction

As a common biometric recognition method, face recognition (FR) has been well applied in the fields of mobile phone unlocking, secure payment, intelligent door lock, and so on. Face recognition uses facial features to identify individuals. However, in these application fields, the information in the face image is lost due to the occlusion problem of the collected face image. Therefore, the lack of some face information will make the effect of face recognition worse. Face recognition is widely used in face image recognition and verification and has attracted extensive attention in recent years. Because FR algorithms are accurate and harmless, they are used in applications such as access control, smart card, information security, and law enforcement [1–3]. People mostly use shallow or deep face recognition learning. The traditional algorithm based on feature extraction uses local or nonlocal facial features to train the model and uses the model to recognize the invisible face image [4, 5]. In addition, in face recognition, the deep learning method is to use the whole face image database to learn and predict the invisible face image without generating specific face features [6]. Most of the current FR approaches assume that multiple images of the same face are available for training their algorithms [7, 8]. In fact, the number of available images of the same face is limited, and the more images used will increase the complexity and processing time of the algorithm. However, when only one sample image can be used for system training, it is difficult to recognize others' faces under different lighting conditions. Therefore, people study FR through local characteristics and achieve more results. Gao proposed a face description and similarity measurement technology for Visual Similarity Retrieval (VSR) in a single model face database, which does not use local operations on isolated points but uses Direction Corner Point (DCP) matching, in which the direction information of connectivity with adjacent points is displayed for similar points [9]. In these feature-based methods, most facial features are usually eyes, nose, and mouth. Because the position and number of facial feature points are not fixed, the number and position of feature vectors can be used to represent different facial features of different faces. The feature points obtained by this method are usually around the main facial features and the individual's special facial features.

In face recognition, the algorithm based on Sparse Representation Classification (SRC) has also been deeply studied [10]. The sparse representation method mainly uses the library image to construct the dictionary and uses the sparse linear combination reconstruction of the dictionary to query the image and complete the matching. However, sparse representation has some shortcomings. For example, sparse representation is applied to each input signal independently regardless of whether the image comes from the same category or not, so some structural information in the image cannot be used. Therefore, some people have also proposed a new facial image representation method, that is, the sequence of forehead, eyes, nose, mouth, and chin, which is similar to sparse representation, that is, based on the similarity between the features extracted from the gallery image area and the features extracted from the query image [11]. In face recognition methods, people use different methods to extract audio fingerprints, such as wavelet transform, Fourier transform, or entropy based for feature matching [12–15]. These algorithms have little interference to environmental noise and volume. The recognition of these local features is a method to create a database, which can not only reduce the information about signals but also retain unique elements, so it is easier to search and find matches in the database.

At the same time, multiple learning has developed rapidly in recent years [16]. Usually, the observation data are located on a low-dimensional submanifold embedded in a high-dimensional environment space [17]. Then, in order to further explore this structure, many manifold learning methods are proposed [18–21]. These algorithms are based on the local invariance principle, and the geometric and topological properties of submanifolds are estimated from the random points sampled from unknown submanifolds. For example, if two data points are very close in the inner manifold, it can be inferred that their representation in any space is also very close [22, 23]. Although designing reliable FR systems requires a lot of work, these systems still face some difficulties, such as when the face looks different under different lighting conditions or facial expressions.

In recent years, in the field of face image recognition, traditional methods based on subspace learning, such as principal component analysis (PCA), linear discriminant analysis (LDA), and its extended versions, are widely used for low-dimensional feature extraction in face recognition [24]. Principal component analysis is an unsupervised method, which aims to obtain a subspace with the largest prediction global variance and maintain the diversity of data [25]. The typical feature face method developed from PCA applies K-L transform to face recognition [26]. The main feature vector of face image usually represents the shape of face, so this method can not only reduce the spatial dimension but also represent the features of face. However, LDA is a monitoring method. This method mainly uses the label information of face image to construct the interclass and intraclass dispersion matrix and makes the face data complete face image recognition in a suitable low-dimensional subspace. The classic fish face based on LDA is also the mainstream face recognition method, which can achieve better classification in face recognition [27].

Recently, a method based on low-rank representation (LRR) has attracted much attention because of its robustness and good performance [28, 29]. This method considers that face data points are located in low-dimensional subspace, and the representation matrix of these data points is low rank. However, the application of LRR and its subsequences is limited because they cannot obtain low-dimensional subspace. The main problem faced by the method based on low-rank representation is how to solve the rank minimization problem. The general solution is to relax it to kernel specification minimization (NNM). Although the kernel norm is a typical convex approximation of the rank minimization problem, the kernel norm minimization makes the solution quite different from the actual value [30]. In view of these situations, this paper presents a face image feature extraction and recognition method based on data dimensionality reduction algorithm. The nonparametric subspace analysis method is used to block the recognition image matrix and preextract the features. After obtaining the low-dimensional new pattern instead of the original image, the face image is digitally processed and recognized. Finally, this method is verified on the AR face database, ORL face database, and XM2VTS face database, and its recognition performance is better than other typical face recognition methods.

## 2. Related Works

### 2.1. Data Dimensionality Reduction

Effective data dimensionality reduction technology can explore the internal structure and relationship of the original data. It can not only eliminate the redundancy between data and simplify data but also greatly improve the computational efficiency, the understandability of data, and the accuracy of the learning algorithm. The adoption of effective dimensionality reduction methods can first compress the data and reduce the amount of storage (eliminating data redundancy will be more effective, large data information is compressed to 50% of the original size, and some can even be compressed to about 20%, greatly saving storage resources). The simplification of data significantly improves the efficiency of calculation, and on this basis, the comprehensibility of data (Intelligence) can be expressed more conveniently. Secondly, the dimension reduction technology is used to make the extracted data features more accurate. It is easy to classify and project to low-dimensional visual space. This step enables us to see the distribution of data in a more intuitive way and internal structure, that is, data visualization.

**Figure 1:**
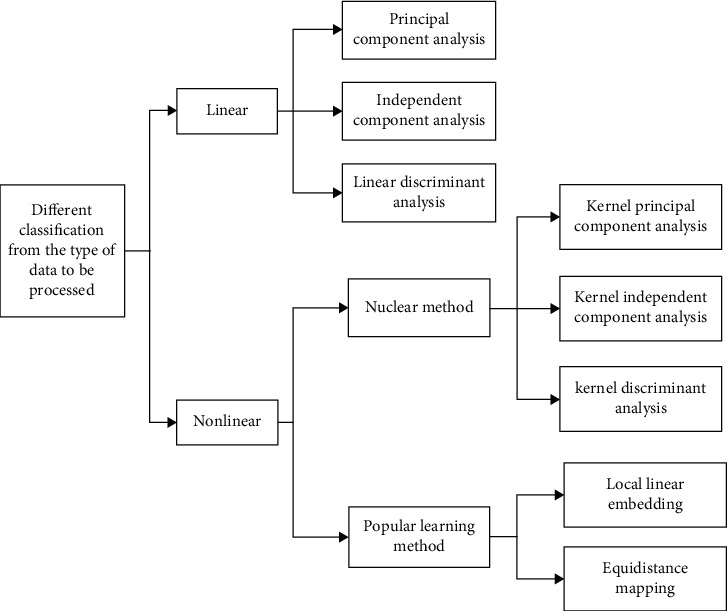
Dimensionality reduction method based on different classification of data attributes to be processed.

Data dimensionality reduction can be divided into two types: feature selection and feature extraction [25]. The former works by finding a subset that can express the original data, while the latter mines new low-dimensional features of the original data. However, these features are not limited to the subset of the original features. Generally speaking, the dimensionality reduction methods used in face recognition are feature extraction. From the perspective of the processed data attributes, data dimensionality reduction can be divided into linear methods and nonlinear methods, as shown in Figure 1.

**Figure 2:**
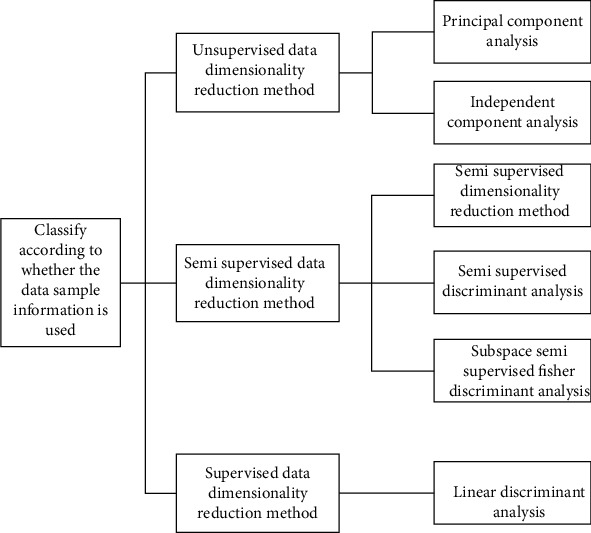
Data dimensionality reduction method classified according to whether the data information to be processed is used.

From the perspective of category information of data to be processed, dimensionality reduction methods can be divided into unsupervised methods, semisupervised methods, and supervised methods. Semisupervised method is the latest research achievement in recent years. It is between nonsupervised and semisupervised, as shown in Figure 2.

### 2.2. Face Recognition

Since the French scientist Francis Galton opened the prelude to human exploration and research on face recognition, after nearly half a century of development, the research field of face recognition has formed two mainstream directions: method based on feature analysis and holistic approach. The method based on feature analysis combines the relative ratio of face reference points with other shape parameters that can be used to describe face features to form the recognition feature vector. The advantage of this method is that it not only retains the topological relationship between face components but also retains a large amount of information about the components themselves. The improved integral projection method is used to extract the 35-dimensional feature vector represented by Euclidean distance, which is then used for pattern classification, including not only the normalized distance and ratio between points but also the two-dimensional topology composed of the corners of the eyes, nose, and mouth. In this way, the overall attributes of the pattern are fully considered. Many experts and scholars have also proposed various improved methods based on this method, such as Fisher face method, elastic graph matching method, RBF network recognition method, hidden Markov model method, and hidden Markov model method.

After years of continuous exploration and research, face recognition has made some achievements in the following directions: face recognition method based on relative difference space, face recognition method based on local, face recognition method based on global, and face recognition method based on subspace.

## 3. Method

### 3.1. Face Recognition and Its Processing

**Figure 3:**
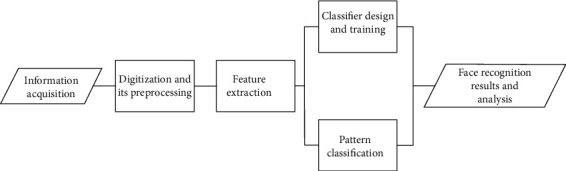
Schematic diagram of face recognition process.

Because faces contain the same features, such as eyes, mouth, and nose, how to effectively recognize different faces is one of the main problems studied by scholars in this field. These features in the face have subtle differences in position, shape, and color. Research shows that the difference between face detection and face recognition is that face detection generalizes face and distinguishes it from nonface. Face recognition must distinguish different faces. Therefore, if face detection is a basic problem, face recognition is a deep-seated problem based on face monitoring. In the design and development of a face recognition system, the problems to be solved include the speed and accuracy of the system from detection to recognition. The system is scalable; that is, it can add more recognition objects. A typical face recognition system is mainly composed of the following five modules, as shown in Figure 3.

Face image information can be obtained through a camera or other digital input devices. The face image information in this paper comes from the commonly used face database. Face image digitization and preprocessing module is an important part of face detection and recognition. In the process of face image acquisition, many factors will lead to the acquisition of images that are not standard enough, for example, the light intensity and brightness of the acquisition environment, the pixel resolution of the acquisition equipment; sometimes, there are phenomena such as insufficient contrast and noise interference in the acquisition. In order to ensure the consistency of faces in the image (face position, face size, image quality, etc.), it is generally necessary to carry out a series of preprocessing operations on the face image. This preprocessing mainly includes the rotation operation, cutting operation, scaling operation, image enhancement operation, geometric normalization processing, and gray normalization processing. In order to obtain the face image in the correct position, it is necessary to straighten the face in the image, and in order to improve the quality of the image, the image is enhanced. By adopting the enhanced image operation, it can not only produce a clearer image visually but also be more conducive to the processing and recognition of the computer, reduce the impact of light on the image, and strengthen the light. In addition, the normalization operation of the image is to obtain a standard face image with the same size and the same gray value.

In terms of feature extraction, firstly, the transformation of the original data is realized through the mapping from face image space to feature space so as to obtain the features that can best reflect the essence of classification. This step provides a reference basis for subsequent work (such as better realization of classification purpose). In the process of feature extraction, we should select appropriate features according to different recognition methods. In a complete face recognition system, feature extraction is the most critical and core part, and it is also one of the greatest difficulties. The effectiveness and stability of the extracted features directly determine the performance and success of the recognition effect.

The design of the classifier is mainly to extract the knowledge of training sequence in unsupervised or semisupervised and supervised ways and obtain the distribution law and class distribution characteristics of the training sequence, so as to provide a basis for subsequent classification decisions. Finally, the pattern classification is used to complete the tasks of face discrimination and classification, and the final discrimination results are obtained.

### 3.2. Feature Extraction Method

In face recognition and processing, the objects to be processed are generally high-dimensional facial makeup images. Due to the sparse distribution of these images in high-dimensional space, it is difficult to classify the images. Therefore, feature selection or extraction methods are often used in face recognition, which can not only improve the execution efficiency of the recognition algorithm but also achieve the purpose of space compression and denoising. The important information of the collected face samples is generally only concentrated in some attributes. The low-dimensional data can be obtained by transforming the high-dimensional data samples so that the most effective features can be extracted. Nonparametric subspace analysis (NSA) can extract these important features from face images [31].

Suppose that there is a training sample set containing *n*(*n*=∑_*i*=1_^*m*^*n*_*i*_) face images, each sample is represented as *Y*_1_, *Y*_2_,…, *Y*_*n*_, they belong to class *m*(*m*_1_, *m*_2_,…, *m*_*k*_), and each sample image is a *h* dimensional column vector. The number of samples of class *im*_*i*_ is expressed as *n*_*i*_.

The total mean vector *ν* of all samples and the sample mean vector *ν*_*i*_ within *m*_*i*_ are expressed, respectively, as follows:

(1) $$\begin{array}{c} \nu =\frac{1}{n}\sum_{i=1}^{m}Y_{i}, \end{array}$$

(2) $$\begin{array}{c} \nu_{i}=\frac{1}{n_{i}}\sum_{k=1}^{n_{i}}Y_{k}, \end{array}$$

where *Y*_*k*_ ∈ *m*_*i*_.

The intraclass scatter matrix of training samples is similar to the interclass scatter matrix *T*_*e*_ in linear discriminant analysis, expressed as follows:

(3) $$\begin{array}{c} T_{e}=\frac{1}{n}\sum_{i=1}^{m}\sum_{j=1}^{n_{i}}\left(Y_{i}^{\left(j\right)}-\nu_{j}\right){\left(Y_{i}^{\left(j\right)}-\nu_{j}\right)}^{T}. \end{array}$$

The interclass dispersion matrix *T*_*f*_^*n*^ of the training sample is expressed as follows:

(4) $$\begin{array}{c} T_{f}^{n}=\frac{1}{n}\overset{m}{\underset{i=1}{\sum }}\overset{m}{\underset{j=1}{\sum }}\overset{n_{i}}{\underset{k=1}{\sum }}\omega \left(i,j,k\right)\left(Y_{i}^{\left(k\right)}-p_{j}\left(Y_{i}^{\left(k\right)}\right)\right){\left(Y_{i}^{\left(k\right)}-p_{j}\left(Y_{i}^{\left(k\right)}\right)\right)}^{T}. \end{array}$$

In equations (3) and (4), *Y*_*i*_^(*j*)^ means the *j*-th image of class *i*(*i*=1,2,…, *m*).

In equation (4), the weight function *ω*(*i*, *j*, *k*) is defined as follows:

(5) $$\begin{array}{c} \omega \left(i,j,k\right)=\frac{\min\left\{l^{\phi }\left(Y_{i}^{\left(k\right)},nn_{j}\left(Y_{i}^{\left(k\right)},i\right)\right),l^{\phi }\left(Y_{i}^{\left(k\right)},nn_{j}\left(Y_{i}^{\left(k\right)},k\right)\right)\right\}}{l^{\phi }\left(Y_{i}^{\left(k\right)},nn_{j}\left(Y_{i}^{\left(k\right)},i\right)\right)+l^{\phi }\left(Y_{i}^{\left(k\right)},nn_{j}\left(Y_{i}^{\left(k\right)},k\right)\right)}. \end{array}$$

In equation (5), *ϕ* is a parameter that varies from zero to infinity. Its function is to control the change speed of distance ratio in weight. *l*(*v*1, *v*2) is the Euclidean distance of vectors *v*1 and *v*2.

*K* nearest neighbor mean *k*_*i*_(*Y*_*i*_^(*j*)^) is defined as follows:

(6) $$\begin{array}{c} k_{i}\left(Y_{i}^{\left(j\right)}\right)=\frac{1}{h}\sum_{q=1}^{h}nn_{q}\left(Y_{i}^{\left(j\right)},i\right). \end{array}$$

In equation (6), *nn*_*q*_(*Y*_*i*_^(*j*)^, *i*) means the nearest neighbor distance from the *q*-th face to the face vector *Y*_*i*_^(*j*)^ in class *i*.

Three conclusions can be obtained from the selection of various parameters in comprehensive equations (3) to (6).

First, if the values of all *ω*(*i*, *j*, *k*) functions are set to integer 1 and *h*=*n*_*i*_, that is, *h* is equal to the number of samples of class *i*, the *K* nearest neighbor mean becomes *ν*_*j*_ (i.e., the sample mean of class *j*).

The interclass scatter matrix of NSA is constructed from the class center, not all training samples. In addition, according to the definition of *K* nearest neighbor mean *k*_*i*_(*Y*_*i*_^(*j*)^), the NSA method can make better use of different types of boundary information.

Substitute equations (3) and (4) into the best criterion function, which is expressed as follows:

(7) $$\begin{array}{c} g\left(w\right)=\frac{\left|w^{T}wT_{f}^{n}\right|}{\left|w^{T}wT_{w}\right|}. \end{array}$$

In equation (7), *w* is the optimal identification projection space. *w* is transformed into a generalized eigenvalue problem.

When *T*_*w*_ is reversible, the characteristic equation is solved, which is as follows:

(8) $$\begin{array}{c} T_{f}^{n}\nu =T_{w}\eta \nu . \end{array}$$

The eigenvector corresponding to the first *h* maximum eigenvalues of equation (8) can be used as the optimal discrimination vector.

By analyzing equations (1) to (8), if the NSA algorithm wants to obtain a high recognition rate, the value of the nearest neighbor *K* is particularly important.

It is assumed that there are *m* categories, which are recorded as *a*_1_, *a*_2_,…, *a*_*m*_. The number of class *i* samples is *k*_*i*_, *B*_*i*_^(*j*)^ is the *j* th *m* of class *i* image matrix *y* × *zi*=1,2, ⋯, *m*, *j*=1,2, ⋯, *k*_*i*_, and the total number of samples is *Y*_sum_=∑_*i*=1_^*m*^*k*_*i*_. The idea of modular 2DPCA is to divide the matrix *B*_*i*_^(*j*)^ into *o* × *p* block matrix, as follows:

(9) $$\begin{array}{c} B_{i}^{\left(j\right)}=\left(\begin{array}{cccc} {\left(a_{i}^{\left(j\right)}\right)}_{1,1} & {\left(a_{i}^{\left(j\right)}\right)}_{1,2} & \ldots & {\left(a_{i}^{\left(j\right)}\right)}_{1,p}\\ {\left(a_{i}^{\left(j\right)}\right)}_{2,1} & {\left(a_{i}^{\left(j\right)}\right)}_{2,2} & \ldots & {\left(a_{i}^{\left(j\right)}\right)}_{2,p}\\ \vdots & \vdots & \vdots & \vdots \\ {\left(a_{i}^{\left(j\right)}\right)}_{o,1} & {\left(a_{i}^{\left(j\right)}\right)}_{o,2} & \ldots & {\left(a_{i}^{\left(j\right)}\right)}_{o,p} \end{array}\right), \end{array}$$

where each image matrix (*B*_*i*_^(*j*)^)_*e*,*f*_(*i*=1,2,…, *m*; *j*=1,2,…, *k*_*i*_; *e*=1,2,…, *o*; *f*=1,2,…, *p*) is matrix *y*_*x*_ × *z*_*x*_(*y*_*x*_ × *o*=*y*, *z*_*x*_ × *p*=*z*).

The overall dispersion matrix of training samples is defined as follows:

(10) $$\begin{array}{c} S_{n}=\frac{1}{n}\sum_{i=1}^{m}\sum_{j=1}^{n_{i}}\sum_{e=1}^{o}\sum_{f=1}^{p}\left({\left(B_{i}^{\left(j\right)}\right)}_{e,f}-\nu \right){\left({\left(B_{i}^{\left(j\right)}\right)}_{e,f}-\nu \right)}^{T}, \end{array}$$

where 1/*n*∑_*i*=1_^*m*^∑_*j*=1_^*n*_*i*_^∑_*e*=1_^*o*^∑_*f*=1_^*p*^(*B*_*i*_^(*j*)^)_*e*,*f*_ is the mean matrix of all training sample submatrixes and the number of all subimages *n*=Mop.

The criterion function is defined as follows:

(11) $$\begin{array}{c} f\left(w\right)=w^{T}wS_{n}. \end{array}$$

Among them, the normalized orthogonal eigenvectors corresponding to the first *n* maximum eigenvalues of *S*_*n*_, *b*_1_, *b*_2_,…, *b*_*n*_ are the optimal projection vector group, and *D* (optimal projection matrix) is expressed as *D*=[*b*_1_, *b*_2_,…, *b*_*n*_]. The characteristic matrix of the training sample projected on *D* is expressed as follows:

(12) $$\begin{array}{c} A_{i}^{\left(j\right)}=\left(\begin{array}{cccc} D^{T}{\left(a_{i}^{\left(j\right)}\right)}_{1,1} & D^{T}{\left(a_{i}^{\left(j\right)}\right)}_{1,2} & \ldots & D^{T}{\left(a_{i}^{\left(j\right)}\right)}_{1,p}\\ D^{T}{\left(a_{i}^{\left(j\right)}\right)}_{2,1} & D^{T}{\left(a_{i}^{\left(j\right)}\right)}_{2,2} & \ldots & D^{T}{\left(a_{i}^{\left(j\right)}\right)}_{2,p}\\ \vdots & \vdots & \vdots & \vdots \\ D^{T}{\left(a_{i}^{\left(j\right)}\right)}_{o,1} & D^{T}{\left(a_{i}^{\left(j\right)}\right)}_{o,2} & \ldots & D^{T}{\left(a_{i}^{\left(j\right)}\right)}_{o,p} \end{array}\right). \end{array}$$

In the process of modular 2DPCA, when the image blocking mode is determined, the number of projection axes (i.e., *n*) determines the dimension of the characteristic matrix (i.e., *A*_*i*_^(*j*)^).

If the value of *n* is too small, the characteristic matrix will lose some favorable discrimination information for later classification. However, if the value of *n* is too large, there will be a lot of redundant information in the characteristic matrix, which will interfere with the dimensionality reduction.

### 3.3. Improved Algorithm

Based on the above algorithm, we could improve the face recognition procession. Let (*y*_*i*_^(*j*)^)_*e*,*f*_=*V*(*A*_*i*_^(*j*)^)_*e*,*f*_; then (*y*_*i*_^(*j*)^)_*e*,*f*_ ∈ *E*^*i*_*k*_*j*_*k*_^, *e*=1,2,…, *o*, *f*=1,2,…, *p*. The mean value of the subimage matrix of the *j*-th image sample of class *i* is calculated as follows:

(13) $$\begin{array}{c} y_{i}^{\left(j\right)}=\frac{1}{op}\sum_{e=1}^{o}\sum_{f=1}^{p}{\left(y_{i}^{\left(j\right)}\right)}_{e,f},\;i=1,2,\ldots ,m,\;j=1,2,\ldots ,k_{i}. \end{array}$$

Note that *nn*_*k*_(*y*_*i*_^(*j*)^, *r*) is the set of *K* nearest neighbors of classes *r* to *y*_*i*_^(*j*)^ and *n*_*r*_(*y*_*i*_^(*j*)^)=1/*k*∑_*n*=1_^*k*^*nn*_*k*_(*y*_*i*_^(*j*)^, *r*) is the mean of *K* nearest neighbors of classes *r* to *y*_*i*_^(*j*)^.

Define the interclass scatter matrix as follows:

(14) $$\begin{array}{c} T_{f}^{n}=\frac{1}{n}\overset{m}{\underset{i=1}{\sum }}\overset{m}{\underset{r=1}{\sum }}\overset{n_{i}}{\underset{k=1}{\sum }}\omega \left(i,j,r\right)\left(y_{i}^{\left(j\right)}-m_{r}\left(y_{i}^{\left(j\right)}\right)\right){\left(y_{i}^{\left(j\right)}-m_{r}\left(y_{i}^{\left(j\right)}\right)\right)}^{T}. \end{array}$$

In equation (14), the weight *ω*(*i*, *j*, *r*) is calculated as follows:

(15) $$\begin{array}{c} \omega \left(i,j,r\right)=\frac{\min\left\{h^{\beta }\left(y_{i}^{\left(j\right)},nn_{k}\left(y_{i}^{\left(j\right)},i\right)\right),h^{\beta }\left(y_{i}^{\left(j\right)},nn_{k}\left(y_{i}^{\left(j\right)},r\right)\right)\right\}}{h^{\beta }\left(y_{i}^{\left(j\right)},nn_{k}\left(y_{i}^{\left(j\right)},i\right)\right)+h^{\beta }\left(y_{i}^{\left(j\right)},nn_{k}\left(y_{i}^{\left(j\right)},r\right)\right)}. \end{array}$$

In equation (15), *β* is a positive parameter in *h*^*β*^(*y*_*i*_^(*j*)^, *nn*_*k*_(*y*_*i*_^(*j*)^, *r*)), which can control the weight change about the distance ratio velocity, and its distance is from *y*_*i*_^(*j*)^ to set *nn*_*k*_(*y*_*i*_^(*j*)^, *r*).

Within class scatter matrix *T*_*w*_ is calculated as follows:

(16) $$\begin{array}{c} T_{w}=\sum_{i=1}^{m}\sum_{j=1}^{n_{i}}\left(y_{i}^{\left(j\right)}-\eta_{i}\right){\left(y_{i}^{\left(j\right)}-\eta_{i}\right)}^{T}. \end{array}$$

In equation (16), *η*_*i*_=1/*n*_*i*_∑_*j*=1_^*n*_*i*_^*y*_*i*_^(*j*)^ is the mean vector of class *i* samples. Therefore, the corresponding criterion function is expressed as follows:

(17) $$\begin{array}{c} G\left(w\right)=\frac{\left|w^{T}wT_{f}^{n}\right|}{\left|w^{T}wT_{w}\right|}. \end{array}$$

The first *T*_*f*_^*n*^*ν*=*T*_*w*_*ην* maximum eigenvalues of the characteristic equation *h* and their corresponding eigenvectors can be used as the optimal discriminant vector.

## 4. Experiment and Analysis

We have carried out relevant experiments on AR database, ORL database, and XM2VTS database to verify that the valuable information of the image is not lost when using this method to extract the low-dimensional features of the image. At the same time, the experiment also compresses this method with other common dimensionality reduction (DR) methods, including LRE [32], LSDP [25], TNNL [33], LPRR [34], and Truncated-LRE [35].

### 4.1. Evaluation Method

The recognition rate and standard deviation are used as evaluation indexes [33]. Firstly, we use the projection matrix obtained by each method to reduce the dimension of the test data set so that the dimension of the test data set changes from 1 to 70 with an interval of 5. Therefore, the recognition rate (rr) of each method in different dimensions can be calculated by the following formula:

(18) $$\begin{array}{c} rr=\frac{\text{sum}\left(pl==al\right)}{n_{t}}, \end{array}$$

where *pl* ∈ *R*^1×*n*_*t*_^ is a set of data prediction labels obtained on the test data set using the DR method, *al* ∈ *R*^1×*n*_*t*_^ is the actual label of a set of data on the test dataset, *n*_*t*_ is the number of samples in the test dataset, and sum() is a function that calculates the number of equal elements at the same position in *pl* and *al*.

In order to avoid randomness, the experiment was repeated five times, and the average recognition rate was calculated. In addition, the stability of the model is evaluated by calculating the standard deviation (SD). The standard deviation can be calculated according to the following formula:

(19) $$\begin{array}{c} s\;d=\sqrt{\frac{\sum_{i=1}^{n}\left(x_{i}-\bar{x}\right)}{n}}, \end{array}$$

where *n* represents the number of repetitions, *x*_*i*_ represents the recognition rate of each experiment, and $\bar{x}$ is the average recognition rate of all experiments.

### 4.2. Experimental Design

In order to evaluate the performance of this method, we verify the effectiveness of this method by evaluating the recognition rate and standard deviation of various methods in different subspaces.

During the experiment, we randomly selected *l* images of a person from each face database for training, and the other images were tested. Moreover, the value of S is adjusted according to the number of people in different databases. *S* = 3, 4, and 5, respectively, represent the AR database, *S* = 4, 5, and 6, respectively, represent the ORL database, and *S* = 5, 6, and 7, respectively, represent the XM2VTS database. Details are shown in Table 1.

### 4.3. Sample Database Description

**Figure 4:**
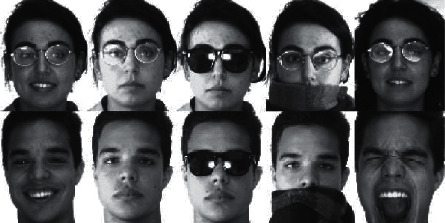
Face image in AR database.

AR database is a large face database established by a computer vision center in Barcelona, Spain, in the 1990s. There are 120 volunteers in this library. Each volunteer has taken 26 images, 3120 in total, of which the proportion of men and women is half, and the age range is between 18 and 45 years. The size of the image is 100 × 80. Each image acquisition environment is different, the light intensity is different, the facial expression is different (open eyes, close eyes, open mouth, shut mouth, laugh, do not laugh, pain, happiness, etc.), and the wearing accessories are also different (scarf, earrings, sunglasses, eyes, etc.). In the experiment, the AR face database is divided into two parts: extracting the training set of human face subspace and a test set to test the performance of the algorithm. The first 10 sheets of each group were used as the training set, and the last 5 sheets were used as the test set. The following is a set of images taken from AR data, as shown in Figure 4.

**Figure 5:**
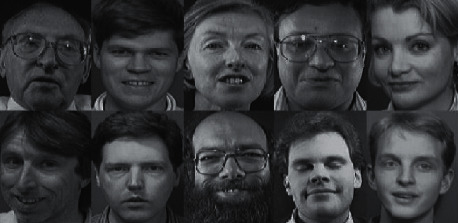
Face image in ORL database.

ORL face database is often selected to test and evaluate the performance of the proposed face recognition algorithm in the world. The shooting time of this database is from 1992 to 1994. There are 400 face images in the ORL face database. All images in this database are 92 × 112 in size, with a gray level of 256. There are 40 volunteers in total. Each volunteer takes 10 images. All volunteers are students and teaching staff. The oldest of the volunteers is 81 years old, the youngest is 18 years old, and most of them are between 20 and 35 years old. There is a large difference in the number of men and women in the database, which is 9 : 1; that is, there are 36 male and 4 female volunteers. The time period of face image acquisition is different, and various details are also different, such as light intensity (strong light and weak light), facial ornaments (with or without glasses or sunglasses, beard, and hair change), and facial expression (smile, serious, open, close, and squint). In the experiment, the ORL face database is divided into two parts: extract the training set of human face subspace and use the first four images of each person as the training set. The last four pictures of each person were used as the test set. The following is a group of photos taken from the ORL face database, as shown in Figure 5.

**Figure 6:**
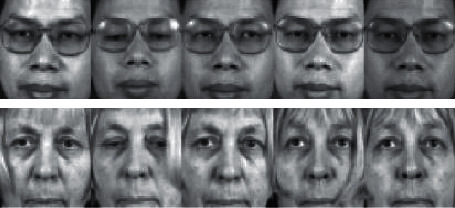
Face image in XM2VTS database.

XM2VTS face database is also one of the commonly used databases in face recognition experiments. There are in total 295 × 8 images or video and audio clips in the library, of which the gray image resolution is 55 × 51; the differences include whether to wear glasses, makeup, expression, and posture. In this experiment, the first five sheets of each group were used as the training set, and the last five sheets were used as the test set. The following is a group of photos taken from the XM2VTS face database, as shown in Figure 6.

### 4.4. Experimental Results

In order to compare the experimental results of each method on the AR face database and objectively reflect the correct recognition rate of images obtained by different methods, the original image matrix is cut into image submatrix sizes 6 × 4 and 12 × 4. After the two blocks are divided, the experimental results of different methods are compared by the recognition rate. The comparison results are shown in Table 2.

Compared with LRE, LSDP, TNNL, LPRR, and Truncated-LRE, the new method is better. Specifically, in the AR database, the results using LRE, LSDP, TNNL, LPRR, and Truncated-LRE methods are 80.46%, 78.69%, 81.07%, 79.63%, and 80.26%, respectively, and the highest recognition rate of the method proposed in this paper is 82.83%.

In order to compare the experimental results of each method on the ORL face database and objectively reflect the correct recognition rate of images obtained by different methods, the original image matrix is cut into image submatrix sizes 8 × 6 and 12 × 6. After the two blocks are divided, the experimental results of different methods are compared by the recognition rate. The comparison results are shown in Table 3.

Compared with LRE, LSDP, TNNL, LPRR, and Truncated-LRE, the new method is better. Specifically, in the ORL database, the results using LRE, LSDP, TNNL, LPRR, and Truncated-LRE methods are 91.46%, 90.69%, 90.07%, 91.63%, and 90.26%, respectively, and the highest recognition rate of the method proposed in this paper is 93.53%.

In order to compare the experimental results of each method on the XM2VTS face database and objectively reflect the correct recognition rate of images obtained by different methods, the original image matrix is cut into image submatrix sizes 10 × 4 and 16 × 4. After the two blocks are divided, the experimental results of different methods are compared by the recognition rate. The comparison results are shown in Table 4.

Compared with LRE, LSDP, TNNL, LPRR, and Truncated-LRE, the new method is better. Specifically, in the XM2VTS database, the results using LRE, LSDP, TNNL, LPRR, and Truncated-LRE methods are 92.43%, 92.59%, 92.17%, 91.35%, and 92.68%, respectively, and the highest recognition rate of the method proposed in this paper is 94.75%.

By comparing the experimental results of this method in different face databases, it can be concluded that this method has an obvious effect on the XM2VTS face database.

## 5. Discussion

According to the experimental statistical results from Tables 2–4, by comparing the correct recognition rates obtained by different methods, it can be seen that this method is better than LRE, LSDP, TNNL, LPRR, and Truncated-LRE methods, but the effect is obvious on XM2VTS face database. In addition, if different methods are used on the same face database, the block method determines the value of the correct recognition rate. When the face databases are the same and the blocking methods are the same, the number of training samples and test samples has a significant impact on the correct recognition rate, but the more training samples, the better the recognition effect.

From the above results, compared with other methods, the methods proposed, LRE, TNNL, and Truncated-LRE, can effectively process the original image and damaged image. This undoubtedly proves the superiority of low-rank regularization in learning the optimal human face subspace. This is mainly because the low-rank representation can accurately capture the subspace structure of the original data. In addition, compared with LRE, the methods proposed, TNNL and Truncated-LRE, have better performance in processing damaged images, which verifies that the method proposed can reduce the negative impact of noise. This is mainly because the TNNL contained in the method proposed and Truncated-LRE replaces the rank function better than the kernel norm in LRE. However, the method proposed has more improvements than TNNL. The key reason is that the method proposed is supervised, and TNNL is not. By adding linear discriminant, the method proposed makes full use of label information. Therefore, the low-dimensional features learned by the method proposed are distinguishable. In conclusion, we can find that the method proposed is the best of these methods. In addition, it also shows the effectiveness of integrating label information and low-rank minimization into the model.

## 6. Conclusion

In the research process of face recognition, feature extraction is not only a key problem but also one of the main difficulties in the development of face recognition. How to effectively extract features directly affects the recognition rate of face image. Aiming at the image processing problem in the process of face recognition, this paper proposes a face image feature extraction and recognition method based on data dimensionality reduction algorithm. The nonparametric subspace analysis method is used to block the recognition image matrix and preextract the features. After obtaining the low-dimensional pattern instead of the original image, the face image is digitally processed and recognized. Finally, through experiments, the method is verified on the AR face database, ORL face database, and XM2VTS face database. According to different methods, its recognition performance is better than other typical face recognition methods.

For two-dimensional face images, the difficulties of face recognition technology still focus on the problems such as illumination intensity and brightness, face expression, and face changes caused by age changes. In recent years, experts and scholars have proposed a face recognition method based on a three-dimensional face model based on these urgent problems. Face recognition method based on 3D face model will be the focus of our research in the next few years [33, 36–38].

## Figures and Tables

**Table 1:** Number of training and testing samples for different databases.

Face databases	S	Training samples	Testing samples
AR database	3	300	500
	4	400	400
	5	500	300

ORL database	4	400	600
	5	500	500
	6	600	400

XM2VTS database	5	500	700
	6	600	600
	7	700	500

**Table 2:** Experimental comparison of different methods on AR face database.

Comparison items	LRE	LSDP	TNNL	LPRR	Truncated-LRE	Method proposed	
						6 × 4	12 × 4
Recognition rate	80.46	78.69	81.07	79.63	80.26	82.29	82.83
Number of misidentified samples	325	357	302	341	335	295	283

**Table 3:** Experimental comparison of different methods on the ORL face database.

Comparison items	LRE	LSDP	TNNL	LPRR	Truncated-LRE	Method proposed	
						8 × 6	12 × 6
Recognition rate	91.46	90.69	90.07	91.63	90.26	93.53	92.95
Number of misidentified samples	19	20	22	18	21	15	17

**Table 4:** Experimental comparison of different methods on XM2VTS face database.

Comparison items	LRE	LSDP	TNNL	LPRR	Truncated-LRE	Method proposed	
						10 × 4	16 × 4
Recognition rate	92.43	92.59	92.17	91.35	92.68	94.13	94.75
Number of misidentified samples	17	16	18	19	15	11	9

## Data Availability

The labeled dataset used to support the findings of this study is available from the corresponding author upon request.
